# Two Misfolding Routes for the Prion Protein around pH 4.5

**DOI:** 10.1371/journal.pcbi.1003057

**Published:** 2013-05-16

**Authors:** Julian Garrec, Ivano Tavernelli, Ursula Rothlisberger

**Affiliations:** 1 Laboratory of Computational Chemistry and Biochemistry - Institute of Chemical Sciences and Engineering, École Polytechnique Fédérale de Lausanne, Lausanne, Switzerland; University of California San Diego, United States of America

## Abstract

Using molecular dynamics simulations, we show that the prion protein (PrP) exhibits a dual behavior, with two possible transition routes, upon protonation of H187 around pH 4.5, which mimics specific conditions encountered in endosomes. Our results suggest a picture in which the protonated imidazole ring of H187 experiences an electrostatic repulsion with the nearby guanidinium group of R136, to which the system responds by pushing either H187 or R136 sidechains away from their native cavities. The regions to which H187 and R136 are linked, namely the C-terminal part of H2 and the loop connecting S1 to H1, respectively, are affected in a different manner depending on which pathway is taken. Specific *in vivo* or *in vitro* conditions, such as the presence of molecular chaperones or a particular experimental setup, may favor one transition pathway over the other, which can result in very different 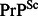 monomers. This has some possible connections with the observation of various fibril morphologies and the outcome of prion strains. In addition, the finding that the interaction of H187 with R136 is a weak point in mammalian PrP is supported by the absence of the 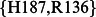 residue pair in non-mammalian species that are known to be resistant to prion diseases.

## Author Summary

Transmissible spongiform encephalopathies, which include the “mad cow” disease and the Creutzfeldt-Jakob disease, are related to the abnormal folding of a host protein termed the prion protein (PrP). Many aspects of the underlying molecular mechanism still remain elusive. Among the hypotheses that have been put forward in the past few years, it has been suggested that PrP could be destabilized by the protonation of a specific residue, H187, when the protein passes through acidic cell organelles. We have modeled PrP at the atomistic level, with the neutral and protonated forms of H187. Our simulations show that the destabilization process can follow two alternative pathways that could lead to different final structures. This discovery may shed some light on one of the most puzzling aspect of prion diseases, the fact that they exhibit various strains encoded in the structure of misfolded PrP. In addition, the atomistic details provided by our model highlight a key interactions partner in the destabilization process, R136. The  residue pair is not present in non-mammalian species that do not develop prion diseases.

## Introduction

The misfolding of the prion protein (PrP), which is a key aspect of transmissible spongiform encephalopathies (TSE), has been the subject of intense research during the past decades. Nonetheless, little is known about the underlying molecular mechanism. One serious hurdle remains the determination of the structure of the resulting misfolded isoform (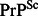) [1]. As a consequence, various 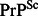 models have been suggested with substantially different packing arrangements and monomer structures, and a consensus about the structure of 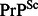 is far from being reached [1]. A particular subject of controversy is about the actual region of PrP that undergoes a deep refolding during the PrP 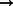 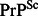 conversion. According to the so-called “spiral” [2] and “
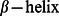
” [3], [4] models, extended 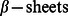 are formed in the N-terminal region and at the beginning of the C-terminal domain up to H1 (H1 is kept intact in the former and is refolded in the latter model). However, it has been recently shown that the H2H3 core is also highly fibrillogenic by itself [5], [6]. Finally, it has also been suggested that 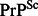 could be entirely refolded in an in-register extended 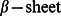 [7].

Many in vitro [5], [8]–[11] and computational [2], [12]–[16] studies have tackled this issue using acidic conditions. They have consistently shown that low pH destabilizes PrP and favors its misfolding. This represents biologically relevant conditions insofar as endosomal organelles, whose typical pH is about 5 but can be as low as 4.3 [17], have been highlighted as possible locations for 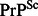 growth [18]–[20]. Importantly, mammalian PrP contain one slightly buried residue, H187, that titrates right in the range of endosomal pH [11], [13]. Several lines of evidence indicate that its protonation [13], or more generally the addition of a positive charge at site 187 [11], destabilizes the protein fold.

**Figure 1:**
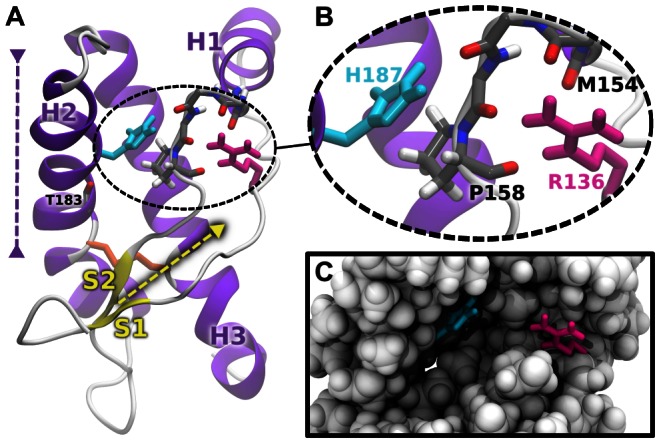
NMR structure of the C-terminal domain of recombinant mouse prion protein (PDB code 1AG2 [21]). (A) Detailed structure showing the secondary structure elements (following the sequence, S1, H1, S2, H2 and H3). The yellow and purple dashed arrows indicate, respectively, the direction of the S1,S2 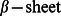 elongation and the partial unraveling of H2 that we observed in our simulations with a protonated H187 (see text). The disulfide bridge is represented in orange sticks. (B) Zoom on the H187,R136 pair and the 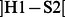 loop (residues 154–158). H187 and R136 are colored in cyan and magenta respectively. Only polar hydrogens and hydrogens of the P158 ring are indicated for clarity. (C) van der Waals surface of the protein arround the solvent-exposed cavities hosting H187 and R136.

Whereas many theoretical studies have been performed on the globular C-terminal domain (residues 121–231 using the numbering of the human sequence) of mouse PrP (mPrP, Fig. 1-A), it is worth noting that the cellular form of PrP (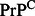) also contains a long unstructured N-terminal tail (residues 23–120) [21]–[29], a glycosylphosphatidyl-inositol (GPI) anchor [30]–[32] and can be mono or diglycosylated [27], [33]. Nevertheless, previous MD simulations have suggested that the structure and dynamics of the globular domain of 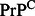 is rather independent of the anchoring to the membrane and the glycosylation [34]. In addition, our previous study of the misfolding propensity of mPrP using extensive REMD simulations [16] has revealed that various 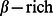 monomers can be formed from the C-terminal domain alone, which is also consistent with the results of Ref. [5], [6].

Here, we have performed microsecond MD simulations of the structured C-terminal domain of mPrP at pH 4.5, which corresponds approximately to the lowest pH value observed in endosomes [17]. To this end, we assigned the protonation state of all titrable residues with the program PROPKA [35] (see also Materials and Methods section). The only buried residue for which the protonation state cannot be uniquely assigned is H187. The quantitative evaluation of its 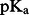 is challenging, because the protonation/deprotonation of a buried residue usually affects the protein structure drastically [36], [37]. Nevertheless, several semi-quantitative estimates of the 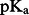 of H187 have been obtained [13], [38] and they all indicate that mPrP coexists in both, neutral and protonated-H187 forms at pH 4.5. Thus we have performed two sets of acidic pH simulations, with H187 in either its neutral or protonated form. It is worth noting that other residues in mPrP also titrate at pH 4.5. However, they are all located at the protein surface, so that their electrostatic effect on the global structure of the protein is much less important than that of H187. Thus, we have considered only one protonation state for these residues (see Materials and Methods section).

Our micorsecond simulations show that the mechanism of mPrP destabilization upon protonation of H187 involves R136 as a key partner (Fig. 1-B,C). There is an electrostatic repulsion between the imidazole ring of the protonated H187 (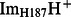) and the guanidinium group of R136 (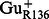), to which the system responds by pushing away either 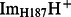 or 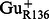. Because R136 and H187 belong to two very different structural regions of the protein, namely the loop connecting S1 to H1 (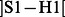) and the C-terminal part of H2 (H2(Cter), Fig. 1-A), the effect on the structure is different depending on which of the two transition routes is taken. It is possible that specific *in vivo* or *in vitro* conditions may favor one route over the other, which could lead to completely different 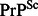 structures. Our findings thus seems to provide some rational to the various conclusions reached by different authors regarding the actual region of the protein that is refolded upon misfolding.

## Results/Discussion

### Conformational changes of the backbone

**Figure 2:**
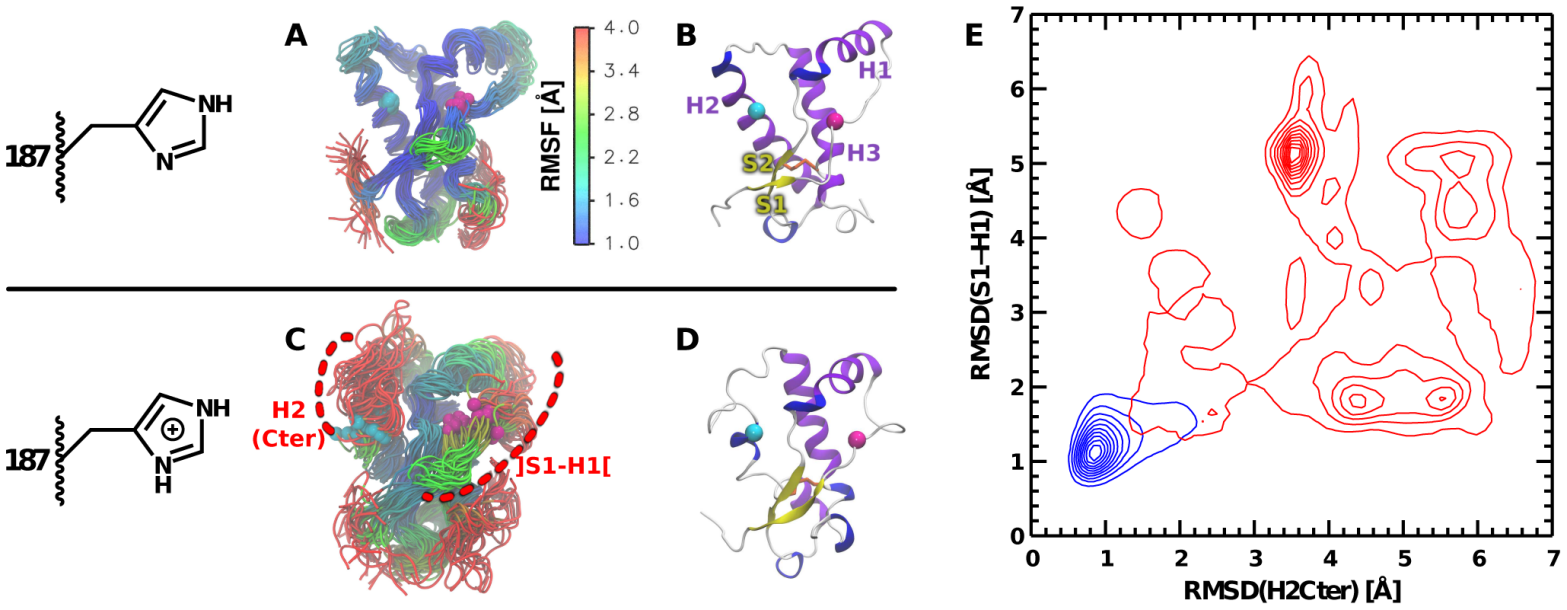
Effect of protonating H187 on the backbone conformation. (A,B) and (C,D) Simulation frames extracted every 50 ns from simulations with a neutral and protonated H187, respectively. The 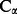 atoms of H187 and R136 are represented with cyan and magenta spheres, respectively. The two regions of high fluctuations when H187 in protonated, H2(Cter) and 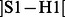, are represented by dashed red arcs in panel C. In panels A,C frames were extracted from two independent simulations of 1 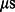 with a neutral H187 and two independent simulations of 1 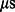 with a protonated H187, respectively, and colored according to the 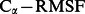. Note that this corresponds to a subset of all our simulation, aimed at providing comparable data between panels A and C. We provide the same representations for each individual simulation in Figure S2. (E) Bivariate distribution of the 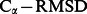 in H2(Cter) and 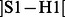. The contour plots are constructed from all the simulations with a neutral (blue) or protonated (red) H187. The reference structure used to compute the RMSD is the average structure calculated from all our simulations with a neutral H187.

Fig. 2 shows the effect of protonating H187 on the backbone of mPrP. The structure is very stable and remains close to the NMR structure when H187 is neutral, whereas simulations with the protonated H187 exhibit important backbone fluctuations and reorganizations. As depicted in Fig. 2-C, these enhanced fluctuations are mainly located in two specific regions of the protein, namely H2(Cter), which hosts H187, and 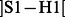.

Fig. 2-E shows that the protonation of H187 induces a drastic change in the free energy surface. The projection of the free energy on the 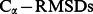 of H2(Cter) and 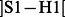 shows a single minimum when H187 is neutral, which corresponds to the native structure of PrP, and a complicated multiple minima landscape when H187 is protonated. The new free energy basins are located 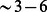 Å away from the native basin, thus corresponding to substantial conformational changes.

The two example snapshots provided in Fig. 2-B,D show that this reorganization is accompanied by a significant modification of the secondary structure of the protein. We will provide a more detailed analysis of the secondary structure changes later in the following sections. For the time being, it is interesting to rationalize how the perturbation that is introduced at one side of the protein (the protonation of H187 located in H2(Cter)) is transmitted through the macromolecule and affects strongly the structure at the opposite side (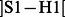).

### Reorganization of charged residues around H187

In order to understand the mechanism by which the protonation of H187 induces the reorganization of the protein structure, it is necessary to have a closer look to the environment of H187 in PrP. It is particularly interesting to focus on nearby charged residues because they are expected to play a major role in the reorganization of the protein when H187 gets a positive charge upon protonation. In the NMR structure of mouse PrP, the closest charged residues are R136, R156, K194, E196 and D202 (Fig. S5-A). R136 is somewhat isolated in terms of proximity with charged residues other than H187 (when protonated), whereas K194, E196, R156, and D202 form a network of salt-bridge interactions. These four latter residues have been pointed out has possible key residues in the misfolding of PrP [13], [39]. As shown in Fig. S5-B, our simulation provide a consistent picture with that of Ref. [13], because the protonation of H187 leads to the disruption of the salt bridge between E196 and R156 and the transient formation of a new salt bridge between the protonated E196 and H187, while a tight salt bridge is maintained between R156 and D202 (K194 is highly solvated, independently of the protonation state of H187, and never interact strongly with E196). Nevertheless, the fact that R136 does not have any close alternative partner makes it more sensitive to the positive electric field created by the protonated H187, as we shall see in the next section.

### Dual response of PrP, 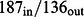 vs 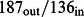


The observation of structural rearrangements in 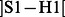, which is located far from H187, has motivated us to perform a thorough analysis of the mobility of each residue in this region. It turns out that R136 is a key partner of H187 in the destabilization of mammalian PrP upon protonation of H187. In the NMR structure of mPrP (Fig. 1), 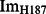 and 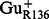 are about 8 Å apart and loop 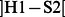 (residues 154–158) is located in between. 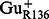 is stabilized by a series of dipole-charge interactions with four peptide bonds while 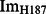 is H-bonded to one carbonyl group and establishes van der Waals contacts with the ring of P158 (Fig. 1-B).

**Figure 3:**
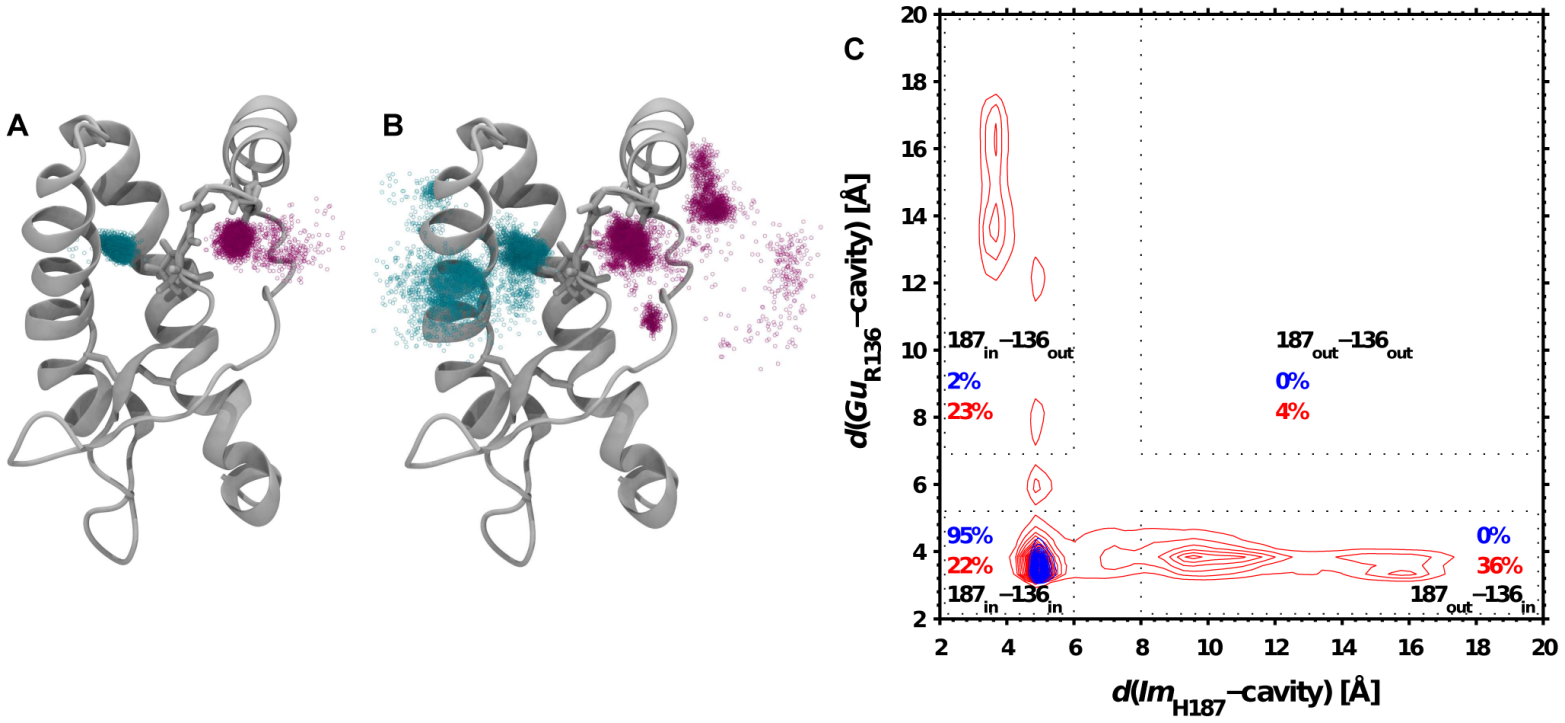
Effect of protonating H187 on the position of 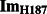 (or 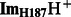) and 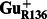. (A) Positions of the 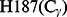 and 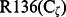 atoms in cyan and magenta, respectively. The positions were extracted every 400 ps from two independent simulations of 1 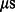 with a neutral H187. Note that this figure is aimed at showing general trends and only represents a subset of all our simulations. We provide the same representations for each individual simulation in Figure S3. (B) Same as (A) but with a protonated H187. (C) Bivariate distribution of the distances between 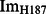 (or 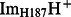) or 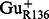 and their respective cavities (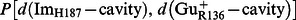). The contour plots are constructed from all the simulations with a neutral (blue) or protonated (red) H187. 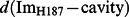 and 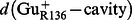 are defined as the distance between the 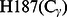 atom and the R156(O) atom and the distance between the 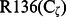 atom and the Y157(O) atom, respectively. The conformational basins (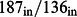, 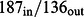 and 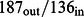, see text) are defined according to the cutoff distances represented by dotted rectangles. The regions with intermediate value are excluded.

Because of the proximity of 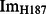 and 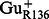 in the native structure of mPrP, the protonation of the former should induce an electrostatic repulsion between the two groups. A discussion of the corresponding energetics is provided in Text S1. Fig. 3 shows the effect of the protonation of H187 on the position of 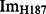 (or 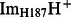) and 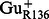. When H187 is neutral, 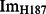 and 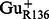 are mostly located in their respective native cavities, whereas they cover a much wider portion of conformational space upon protonation of H187. We define four conformational states according to the position of 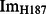 (or 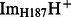) and 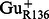 inside or outside their respective native cavities. To do so we consider the bivariate histogram of the distances between 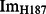 (or 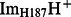) and 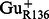 from their respective cavities (Fig. 3-C). The conformational state in which both groups stay close to their original location will be termed 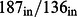, and we define states 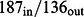 and 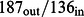 according to the departure of 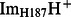 or 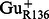, respectively. Interestingly, the 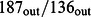 state is almost not populated. The picture that is the most consistent with these data is that PrP exhibits a dual response to the protonation of H187, by pushing away either 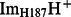 or 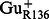 (but not both at the same time), thus decreasing the electrostatic repulsion between them. Because H187 and R136 are attached to H2(Cter) and 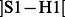, respectively (Fig. 1-A,B), the local reorganization of either 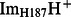 or 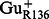 affects the global structure of these two regions (Fig. 2). We stress that, once H187 is protonated, the dynamics of the system proceeds smoothly through a series of locally thermalized states giving rise, in a reproducible way, to either the 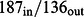 or 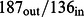 state. Fig. S6 and S7 show that 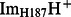 and 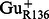 remain in their native pockets during at least 100 ns before one of the two moves out.

A similar electrostatic repulsion can be expected for the H187R mutation, for which the positive charge of the introduced arginine has been suggested to destabilize the overall fold of human PrP [11], [40], [41]. An interesting aspect of this finding is that none of the non-mammalian PrP exhibit this specific 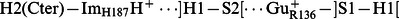 spatial arrangement (Fig. S4). In other words, these non-mammalian proteins do not have this pH-sensitive “weak point” in there structure and this probably explains the fact that non-mammalian species do not exhibit TSEs.

Due to the buried character of H187 and the fact that its protonation induces a substantial modification of the protein structure, the quantitative evaluation of its 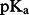 (and the corresponding contributions of other residues) during the misfolding is challenging [36], [37]. Nevertheless, PROPKA calculations [35] provide physically sound estimates that can help to rationalize the underlying physics. Such calculations for representative snapshots of our simulations are provided in Fig. S8. The 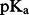 of H187 is systematically shifted up as soon as the protein starts to misfold, independently of the pathway (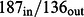 vs 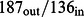) that is taken. This is in agreement with the fact that the proximity with the positive charge of 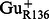 in the native structure of mPrP induces a down-shift of the 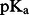 of H187 (this is supported by the fact that our PROPKA calculations report R136 as a key residue in the electrostatic environment of H187, see the corresponding PROPKA output file for a representative 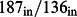 structure in Dataset S1). As soon as 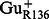 moves out of its cavity (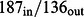 state) the electrostatic repulsion between 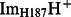 and 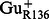 decreases and the protonated form of H187 becomes much more stable (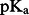 shifted up). When the protein adopts a 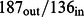 state, 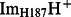 is much more solvated by water and the 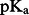 of H187 approaches the corresponding value in water (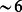).

### S1,S2 elongation

**Figure 4:**
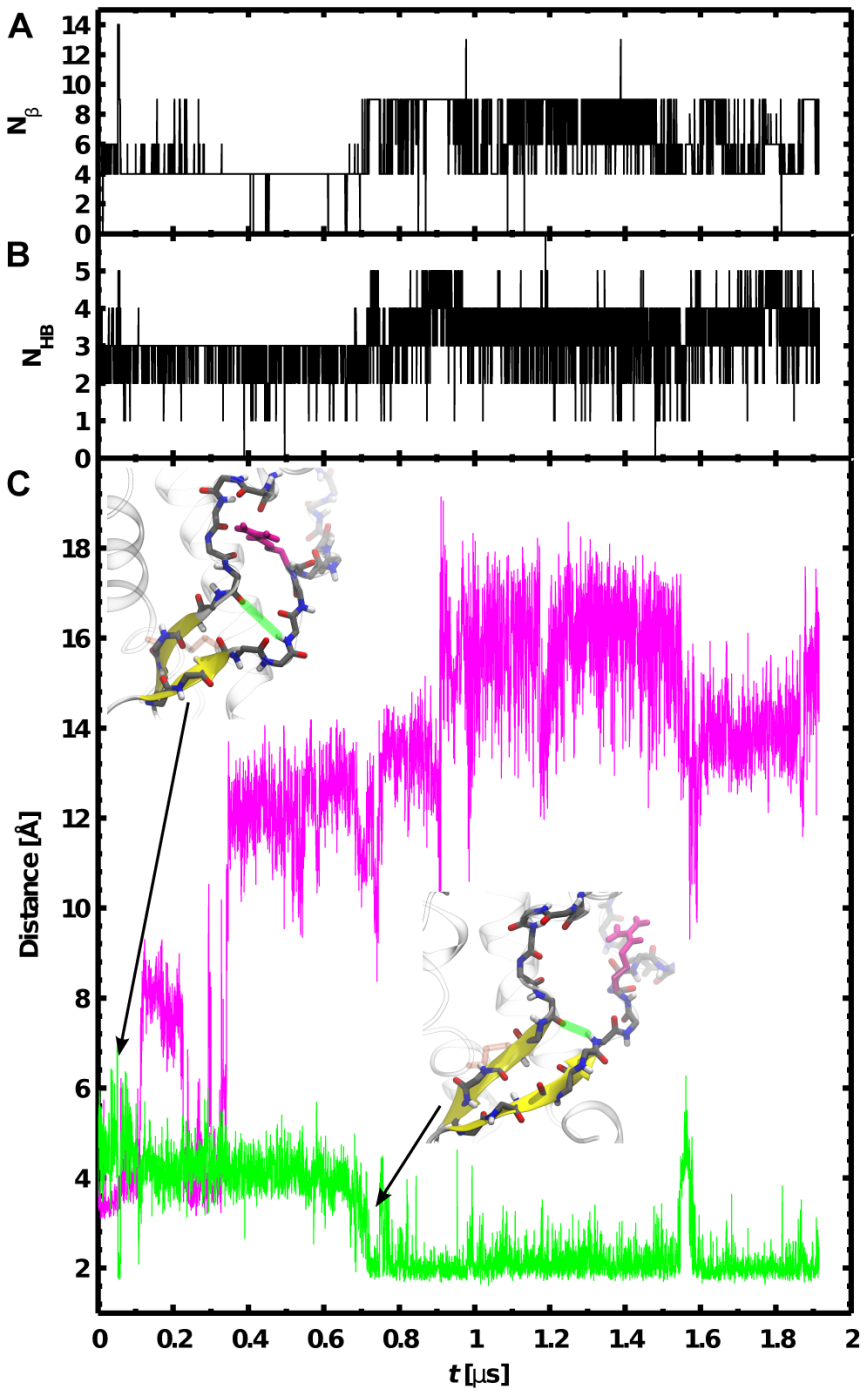
Link between the motion of 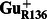 toward bulk water and the elongation of S1,S2. The data are taken from a simulation in which a 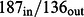 state is formed permanently. (A) Time evolution of the number of residues in a 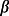 conformation. (B) Time evolution of the number of backbone hydrogen bond between 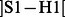 and 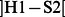 (the backbone atoms of these two loops are represented with sticks in the two MD snapshots shown in panel C). (C) Time series of 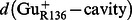 (same definition as in Fig. 3) and the distance between the extremities of the two strands (represented with a thick green line in the two MD snapshots).

The positioning of 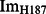 (or 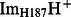) and 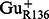 has a strong influence on the length of the S1,S2 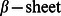, as shown in Table 1. Typically, both 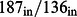 and 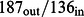 states correspond to structures with a short native 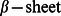, while the 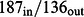 state is characterized by the preference of an elongated 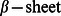. This is illustrated by the simulation depicted in Fig. 4. At the beginning of the simulation, the protein is in its native conformation. As depicted in the insets of Fig. 4, the native location of 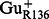 at 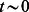 is a key aspect of the protein fold because it forms a sort of “clip” that forces the 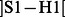 backbone to remain packed against the rest of the protein (Fig. 1-A) in a specific conformation. The permanent departure of 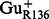 out of its cavity at 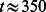 ns induces an important release of 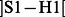 backbone constraint and the system is consequently more prone to reorganize in this region. Then the system relaxes during about 400 ns, and 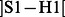 and 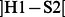 come close together. The number of hydrogen bonds between the two strands increases concomitantly and the 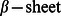 elongates (Fig. 4-A,B).

**Table 1:** Number of short/long 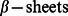.

	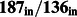 b	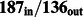 c	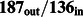 c
Population of short 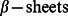 [%] 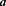	93.2	65.3	99.0
Population of long 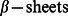 [%]^ a^	6.8	34.7	1.0

a The number of residues in a 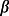 conformation in the structures of mammalian PrP taken in the PDB is either 4 or 6. Hence we define a short and a long 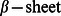 as a 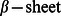 with a number of residues 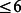 or 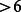, respectively. The populations are in % of the corresponding cluster.

b Cluster extracted from the simulations with a neutral H187.

c Cluster extracted from the simulations with a protonated H187.

### H2 unraveling

**Figure 5:**
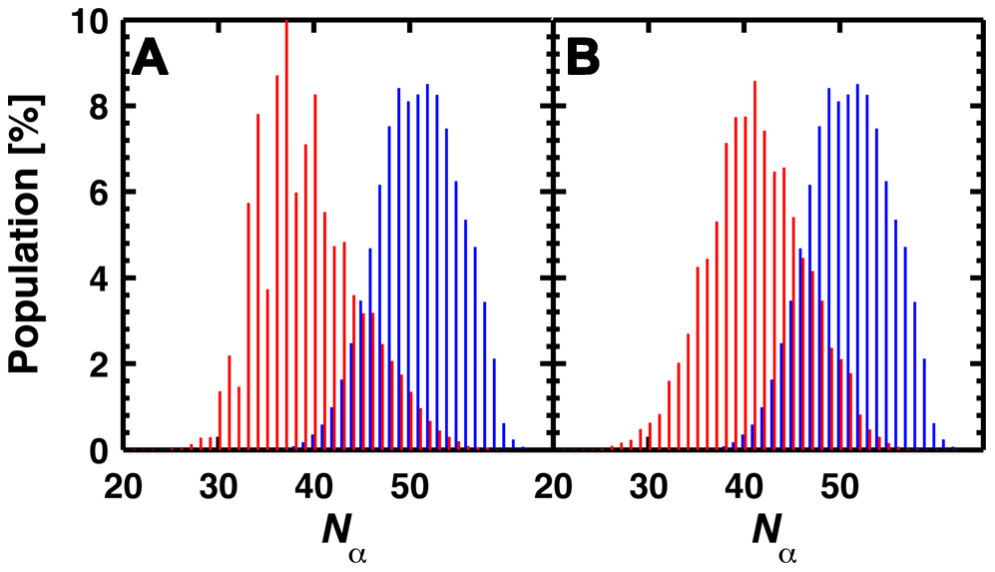
Effect of the 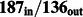 and 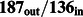 conformations on the number of helical residues (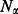). (A) Distribution of 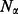 in the 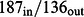 cluster extracted from simulations with a protonated H187 (red) compared to the 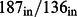 cluster extracted from simulations with a neutral H187 (blue). (B) Distribution of 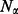 in the 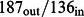 state extracted from simulations with a protonated H187 (red) compared to the 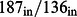 state extracted from simulations with a neutral H187 (blue). The populations are in % of the corresponding cluster.

As shown in Fig. 5, both 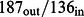 and 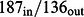 states are characterized by an unraveling of H2(Cter). However, the underlying mechanisms (and the corresponding transition pathways) differ substantially. The portion of the helix that undergoes an unraveling is represented by a dashed purple arrow in Fig. 1-A (see also the example snapshot depicted in Fig. 2-D).

**Figure 6:**
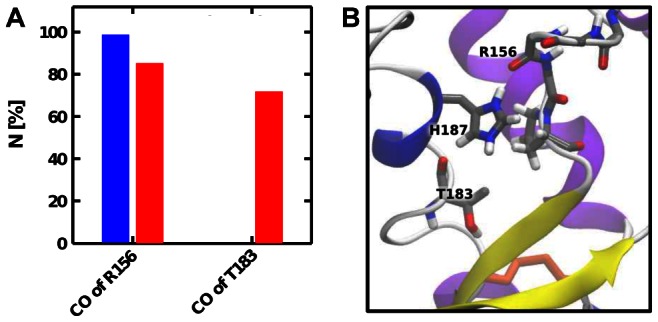
Polar interactions of 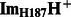 in its cavity when the system is in the 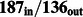 state. (A) Number of contacts (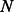) between 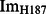 (or 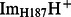) and the CO groups of R156 and T183 in the 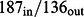 cluster extracted from simulations with a protonated H187 (red) compared to the 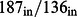 cluster extracted from simulations with a neutral H187 (blue). (B) Example snapshot showing 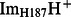 interacting with both CO groups when the systems is in the 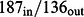 state. Note that 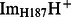 can interact with R156(CO) alone, T183(CO) alone, or both.

The departure of 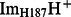 (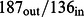 conformation) out of its cavity obviously destabilizes H2 because the helix looses a key tertiary contact with loop 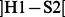 (Fig. 1-A). The unraveling of H2(Cter) in the 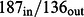 state has its roots in the polar interactions of 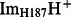 with the nearby residues. A closer look to the shape of the 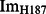 cavity (Fig. 1) reveals that it is a narrow groove at the bottom of which lies the carbonyl group of T183. The contact analysis shown in Fig. 6 reveals that the neutral 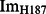 is H-bonded to R156 only, consistent with the NMR structure of mPrP [21], whereas new contacts are formed with the CO group of T183 when H187 is protonated. A key aspect of these extra contacts is that they involve not only the 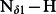 group of H187, but also the 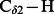 group. They reflect dipole-charge interactions between the extra positive charge of 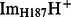 and the dipole moments of the 156–157 and 182–183 peptide bonds. In other words, the imidazole ring can take two conformations around the 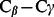 bond and still maintain a significant interaction with one of the two nearby backbone CO groups, which results in four stable conformations inside the pocket.

The formation of new contacts between 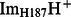 and T183(CO) has two effects that explain the loss of helical character in H2(Cter). First of all, it weakens the tertiary contact between H2(Cter) and 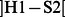. Second, the native intra-helix H-bond between T183(CO) and H187(NH) is lost. The tighter the interaction between 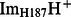 and T183(CO) the weaker the local stability of H2.

### Concluding remarks

In this paper we have shown that the protonation of H187 in mPrP at pH 4.5, which corresponds approximately to the lowest pH observed in endosomes [17], leads to extensive conformational changes on the microsecond time scale. The picture that emerges from our simulations is that the protonation of H187 leads to an electrostatic repulsion between the positive charges of 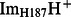 and 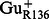, which results in conformational transitions in the regions to which H187 and R136 are linked, namely H2(Cter) and 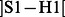 respectively.

Our findings hence highlight two possible routes for PrP misfolding with either the unraveling of H2(Cter) alone (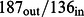 route) or the unraveling of H2(Cter) with simultaneous elongation of S1,S2 (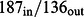 route). This dual behavior seems to reconcile the various observations and proposals that have been made regarding the actual PrP region that undergoes a deep refolding upon conversion to 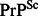 [2]–[5]. It is indeed possible that a particular computational or experimental setup favors one of the 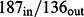 or 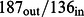 substates at the beginning of the misfolding process. Such conformational shift could be assisted *in vivo* by molecular chaperones such as polyanionic molecules [1], [42]. This variability in misfolding pathways may also be connected to the fact that prion exhibits a variety of strains, because it is believed that changes in conformations of 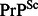 encodes for strain properties [30], [43], [44].

Finally, it is interesting to note that the 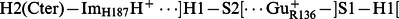 pattern is not present in those non-mammalian species who are known to resist to TSEs. This is a possible explanation for the observed resistance to TSEs in these species.

## Materials and Methods

### Initial structure and protonation state

All simulations were started from the NMR structure of mPrP published by Riek *et al.* (PDB code 1AG2). We aimed at modeling mPrP with a neutral or protonated H187 at pH 4.5.

The protonation state of titrable residues apart from H187 was first estimated from PROPKA [35] calculations. The protonation state of most of them can be determined without ambiguity. All buried or semi-buried residues other than H187 are all aspartic or glutamic acids whose side chains are hydrogen-bonded to other groups in the protein. This has the effect to shift up their 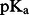 above the typical values of 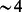 that they adopt in water, i.e. significantly above the pH we want to model. Hence they are expected to be protonated. The solvent-exposed histidines are expected to exhibit a 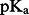 of 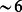 so they can be considered protonated at a pH of 4.5. The remaining solvent-exposed aspartic or glutamic acids are more ambiguous because their 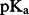 is close to the pH we want to model. Nevertheless, their solvent-exposed character makes them much less important for the global fold of the protein. We chose their protonation state according to the 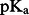 estimated with PROPKA [35]. The relevance of this choice was verified *a posteriori* by observing that the fold of the protein is very well conserved over microsecond simulations with a neutral H187.

### Simulation setup

Two topologies (one with H187 neutral and one with H187 protonated) were built with the GROMACS 4.0.7 [45]–[47] suite of programs. For each of them, the protein was immersed in a rhombic dodecahedral water box. The size of the box was chosen so that the distance between the protein and the edge of the box was 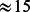 Å. The system was neutralized by adding 2 or 3 chloride counterions (depending on the protonation state of H187). The resulting system contained about 30000 atoms.

The AMBER99SB force field [48] was used to describe the protein and the TIP3P model [49] was employed for the water molecules. The force field was included in GROMACS thanks to the ports provided by Sorin and coworkers [50], [51]. The particle mesh Ewald method [52] together with a Fourier grid spacing of 1 Å and a cutoff of 12 Å was used to treat long-range electrostatic interactions. A cutoff of 12 Å was used for van der Waals interactions. The water box was first relaxed by means of NpT simulations with restraints applied to the positions of the heavy atoms of the protein. Then the system was optimized in a series of energy minimization runs in which the restraints on the protein were progressively removed. Finally, we run eight simulations with a time step of 2 fs. Three and five of them were conducted with a neutral or protonated H187, respectively.

Each simulation was initiated with a set of velocities taken at random from a Maxwell-Boltzmann distribution corresponding to a temperature of 10 K. Then the system was heated up to 300 K in 300 ps using two Berendsen thermostats [53] (one for the protein and one for the solvent) with a relaxation time of 0.1 ps each. The simulation was prolonged for 100 ps and the Berendsen barostat with a relaxation time of 2 ps was switched on during 100 ps. Finally, we switched to production phase using a Nose-Hoover [54], [55] thermostat and a Parrinello-Rahman barostat [56] with relaxation times of 0.5 and 10.0 ps, respectively. The total simulation lengths were 1.9, 1.3 and 1.6 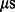 for simulations with a neutral H187, and 1.9, 1.5, 1.6, 1.2 and 1.2 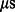 for simulations with a protonated H187. The 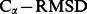 plot of each simulation is provided in Figure S1.

### Molecular visualization and analysis

All the representations were done with the program VMD [57]. Secondary structure assignments were done using the STRIDE algorithm [58].

## Supporting Information

Text S1
**Energetics of the** 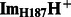 **-** 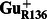 **ion pair in the native structure of mPrP.** Estimation of the electrostatic interaction between 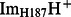 and 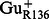 using a Coulomb-type expression [59]–[61] and the typical dielectric constant inside a protein [59], [61], [62]. Discussion of the strength of this interaction and its implication on the protein stability.(PDF)

Dataset S1
**PROPKA** [**35**] **output file obtained from a representative structure of a** 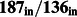 **state (same structure as Fig. S8-A.).**
(TXT)

Dataset S2
**PROPKA** [**35**] **output file obtained from a representative structure of a** 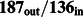 **state (same structure as Fig. S8-B.).**
(TXT)

Dataset S3
**PROPKA** [**35**] **output file obtained from a representative structure of a** 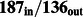 **state (same structure as Fig. S8-C.).**
(TXT)

Figure S1

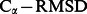
 **.** Each panel corresponds to one individual simulation, differing by the initial velocities extracted at random from a Maxwell-Boltzmann distribution. (A–C) Three individual simulations in which H187 is neutral. (D–H) Five individual simulations in which H187 is protonated.(TIF)

Figure S2
**Backbone fluctuations.** Each panel corresponds to one individual simulation, differing by the initial velocities extracted at random from a Maxwell-Boltzmann distribution. (A–C) Three individual simulations in which H187 is neutral. (D–H) Five individual simulations in which H187 is protonated. Simulation frames are extracted every 50 ns. The 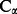 atoms of H187 and R136 are represented with cyan and magenta spheres, respectively. The backbone is colored according to the 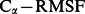 (same scale as Fig. 2).(TIF)

Figure S3
**Position of** 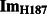 **(or** 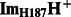 **) and** 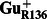 **.** The positions of the 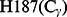 and 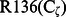 atoms are represented by cyan and magenta spheres, respectively. Each panel corresponds to one individual simulation, differing by the initial velocities extracted at random from a Maxwell-Boltzmann distribution. (A–C) Three individual simulations in which H187 is neutral. (D–H) Five individual simulations in which H187 is protonated. Simulation frames are extracted every 400 ps.(TIF)

Figure S4
**Mammalian VS non-mammalian species.** The upper panel of the figure shows the sequence alignment in the H2(Cter) region. H187 is represented in cyan in the sequence and is conserved throughout all mammalian PrP. The lower panel represents the charged residues in examples of mammalian (mouse) and non-mammalian (chicken) PrP. The geometric center of positively and negatively charged groups are represented by blue and red opaque spheres, respectively. The two transparent spheres in cyan and magenta correspond to the position of 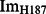 and 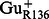 in mPrP, respectively. Sequence and structural alignments were done with the MultiSeq plugin [63] implemented in VMD [57].(TIF)

Figure S5
**Charged residues around** 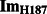 **.** (A) Relative positioning of the residues. All charged groups around 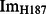 fall approximately within the same range of distance, which is represented by a transparent sphere of 8 Å diameter centered on the geometric center of 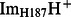. (B) Population of salt bridges in our simulations with a neutral (blue) or protonated (red) H187.(TIF)

Figure S6
**Distance of** 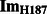 **(or** 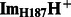 **) from its cavity as a function of time.** (A–C) Three individual simulations in which H187 is neutral. (D–H) Five individual simulations in which H187 is protonated. The distance 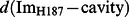 is defined as in Fig. 3-C. The magenta box represents the cutoffs used in Fig. 3-C to define 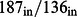, 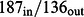, 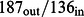 and 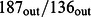 states.(TIF)

Figure S7
**Distance of** 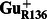 **from its cavity as a function of time.** (A–C) Three individual simulations in which H187 is neutral. (D–H) Five individual simulations in which H187 is protonated. The distance 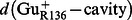 is defined as in Fig. 3-C. The magenta box represents the cutoffs used in Fig. 3-C to define 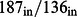, 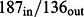, 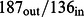 and 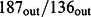 states.(TIF)

Figure S8

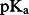
 **of H187 as a function of the relative positioning of** 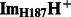 **and** 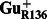 **.** Representative snapshots of (A) a 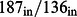 state (equilibrated structure before 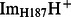 or 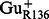 moves out of its cavity), (B) a 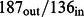 state, and (C) a 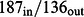 state. Water molecules that are within 3 Å of 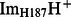 or 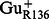 are represented in cyan and magenta, respectively. The number close to H187 in each panel indicates the 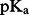 of this residue, as estimated by PROPKA [35] from the corresponding structure. The calculations were performed using the PDB2PQR software [64], [65]. We also provide the PROPKA output files corresponding to panels (A), (B) and (C) in Dataset S1, S2 and S3, respectively.(TIF)
