# Supplementary material for: Two Misfolding Routes for the Prion Protein around pH 4.5
Source: PLoS Comput Biol. 2013 May 16;9(5):e1003057. doi: 10.1371/journal.pcbi.1003057 (PMC3656106; doi:10.1371/journal.pcbi.1003057)
Supplement: Text S1 — Energetics of the - ion pair in the native structure of mPrP. Estimation of the electrostatic interaction between and using a Coulomb-type expression [59]–[61] and the typical dielectric constant inside a protein [59], [61], [62]. Discussion of the strength of this interaction and its implication on the protein stability. (PDF) [file pcbi.1003057.s001.pdf]

Text S1. Energetics of the $\mathrm{Im_{H187}H^+}$-$\mathrm{Gu^+_{R136}}$ ion pair in the native structure of mPrP

We estimated the strength of the electrostatic repulsion between $\mathrm{Im_{H187}H^+}$ and $\mathrm{Gu^+_{R136}}$ in the native structure of mPrP. As shown in Fig. S6 and S7, once H187 is protonated, $\mathrm{Im_{H187}H^+}$ and $\mathrm{Gu^+_{R136}}$ remain in their native pockets during at least 100 ns before one of the two moves out. This speaks for a subtle local perturbation which is then propagated to specific parts of the system. To quantify the strength of this repulsion, we used the Coulomb-type expression [1]:

$$\Delta G_{qq}(r) = 332\frac{q(\mathrm{Im_{H187}H^+})q(\mathrm{Gu^+_{R136}})}{\epsilon(r)r}$$

where the $q(i)$ are the charges of the corresponding groups (+1 here), r is the distance separating them in Å (8 Å here), and $\epsilon(r)$ is an effective distance-dependent dielectric function [2,3]. This expression can be simplified further by using the fact that the dielectric constant typically ranges between 20 and 40 in protein interiors [1,3,4]. This gives an estimates of $\sim$ 1–2 kcal/mol for the electrostatic repulsion between $\mathrm{Im_{H187}H^+}$ and $\mathrm{Gu^+_{R136}}$ in the native structure of mPrP. This is significant compared to the typical value of the folding energy of a protein, and hence has some substantial consequences on the protein structure after a certain amount of time (Fig. S6 and S7).

References

1. Warshel A, Russell S, Churg A (1984) Macroscopic models for studies of electrostatic interactions in proteins - limitations and applicability. Proceedings of the National Academy of Sciences of the United States of America-Biological Sciences 81: 4785–4789.

2. Schutz C, Warshel A (2001) What are the dielectric "constants" of proteins and how to validate electrostatic models? Proteins Struct Funct Genet 44: 400–417.

3. Warshel A, Sharma PK, Kato M, Parson WW (2006) Modeling electrostatic effects in proteins. Biochim Biophys Acta 1764: 1647–1676.

4. Roca M, Messer B, Warshel A (2007) Electrostatic contributions to protein stability and folding energy. FEBS Lett 581: 2065–2071.
