# Supplementary figures and images for: Two Misfolding Routes for the Prion Protein around pH 4.5
Source: PLoS Comput Biol. 2013 May 16;9(5):e1003057. doi: 10.1371/journal.pcbi.1003057 (PMC3656106; doi:10.1371/journal.pcbi.1003057)

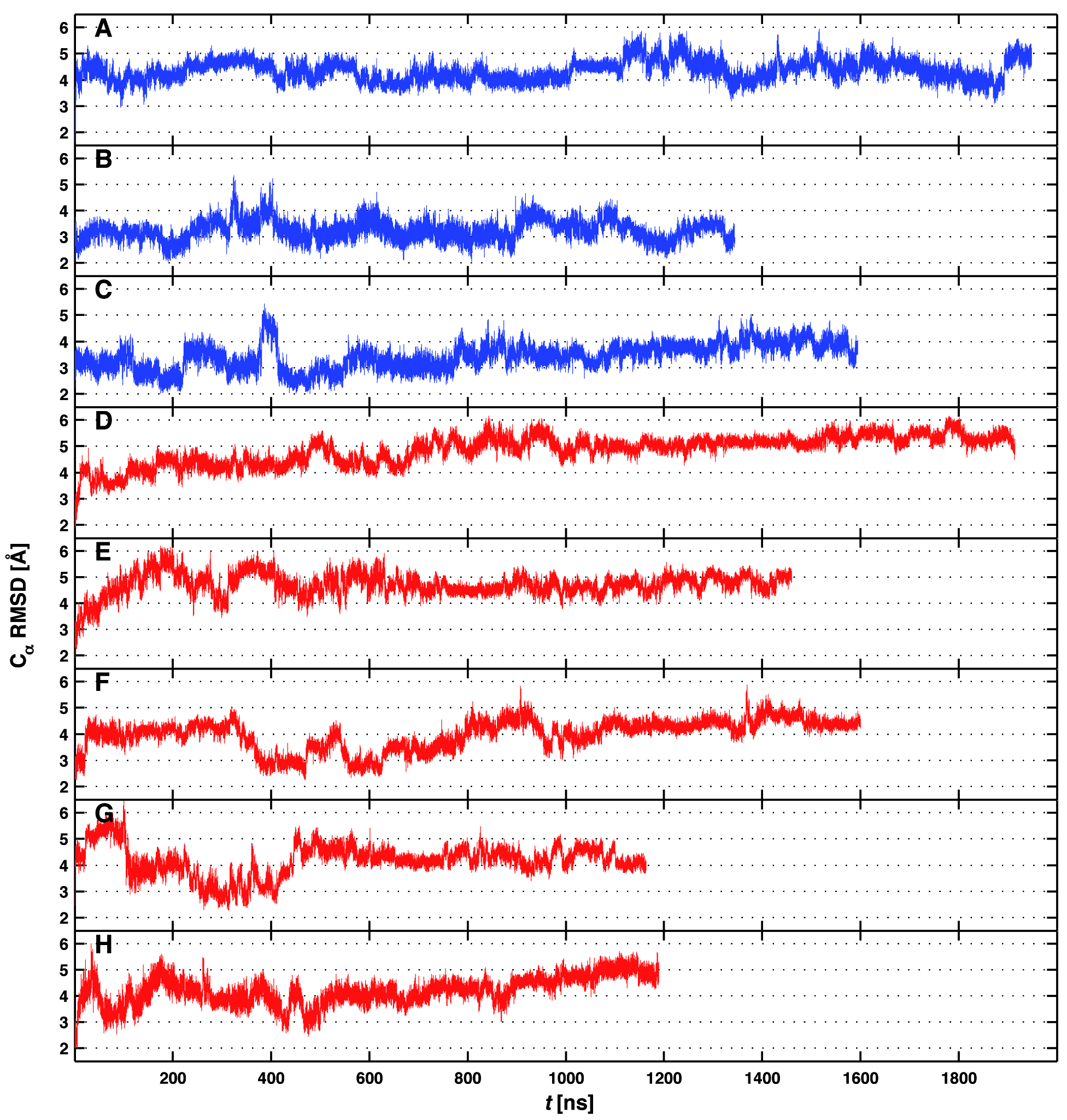

Supplement: Figure S1 — . Each panel corresponds to one individual simulation, differing by the initial velocities extracted at random from a Maxwell-Boltzmann distribution. (A–C) Three individual simulations in which H187 is neutral. (D–H) Five individual simulations in which H187 is protonated. (TIF) [file pcbi.1003057.s005.tif]

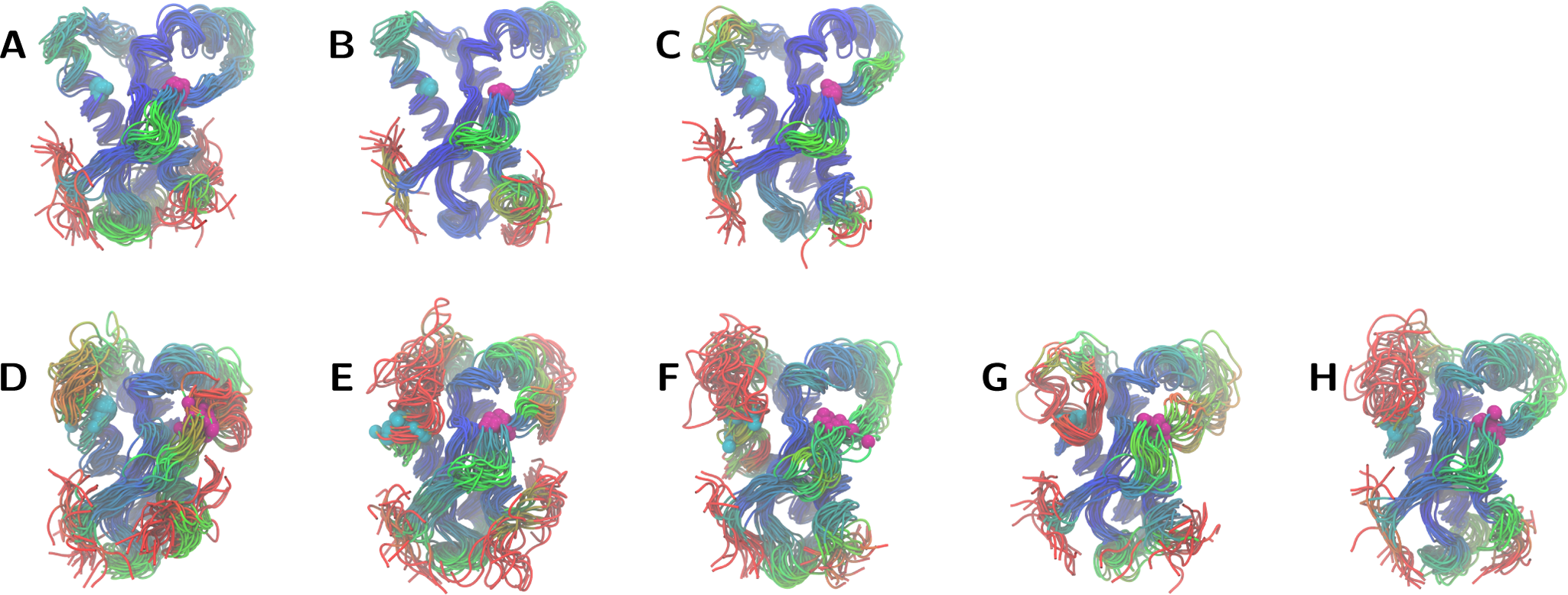

Supplement: Figure S2 — Backbone fluctuations. Each panel corresponds to one individual simulation, differing by the initial velocities extracted at random from a Maxwell-Boltzmann distribution. (A–C) Three individual simulations in which H187 is neutral. (D–H) Five individual simulations in which H187 is protonated. Simulation frames are extracted every 50 ns. The atoms of H187 and R136 are represented with cyan and magenta spheres, respectively. The backbone is colored according to the (same scale as Fig. 2). (TIF) [file pcbi.1003057.s006.tif]

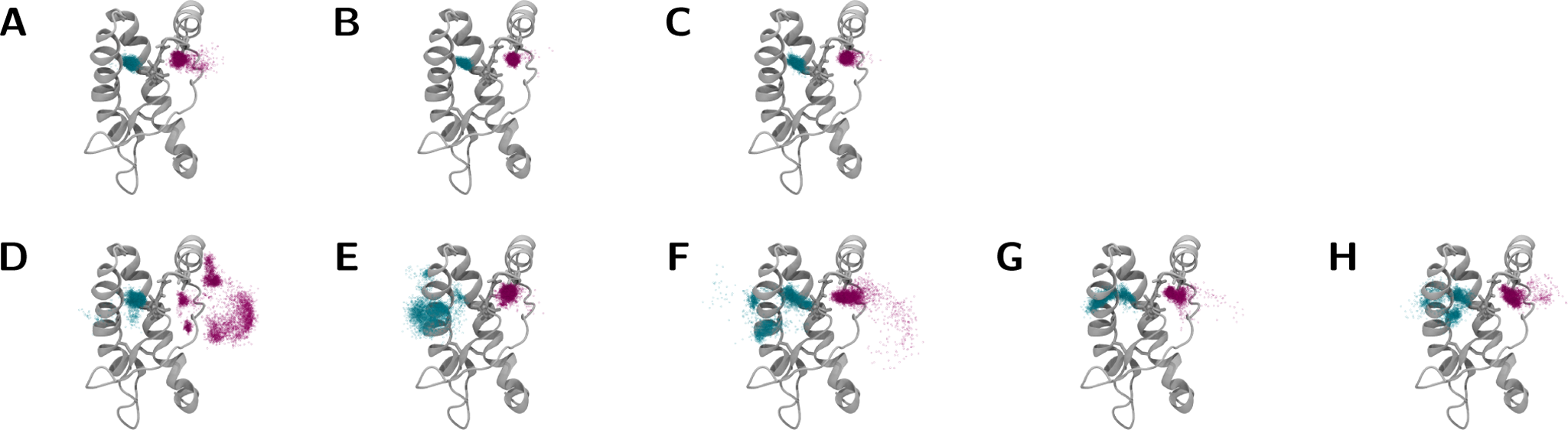

Supplement: Figure S3 — Position of (or ) and . The positions of the and atoms are represented by cyan and magenta spheres, respectively. Each panel corresponds to one individual simulation, differing by the initial velocities extracted at random from a Maxwell-Boltzmann distribution. (A–C) Three individual simulations in which H187 is neutral. (D–H) Five individual simulations in which H187 is protonated. Simulation frames are extracted every 400 ps. (TIF) [file pcbi.1003057.s007.tif]

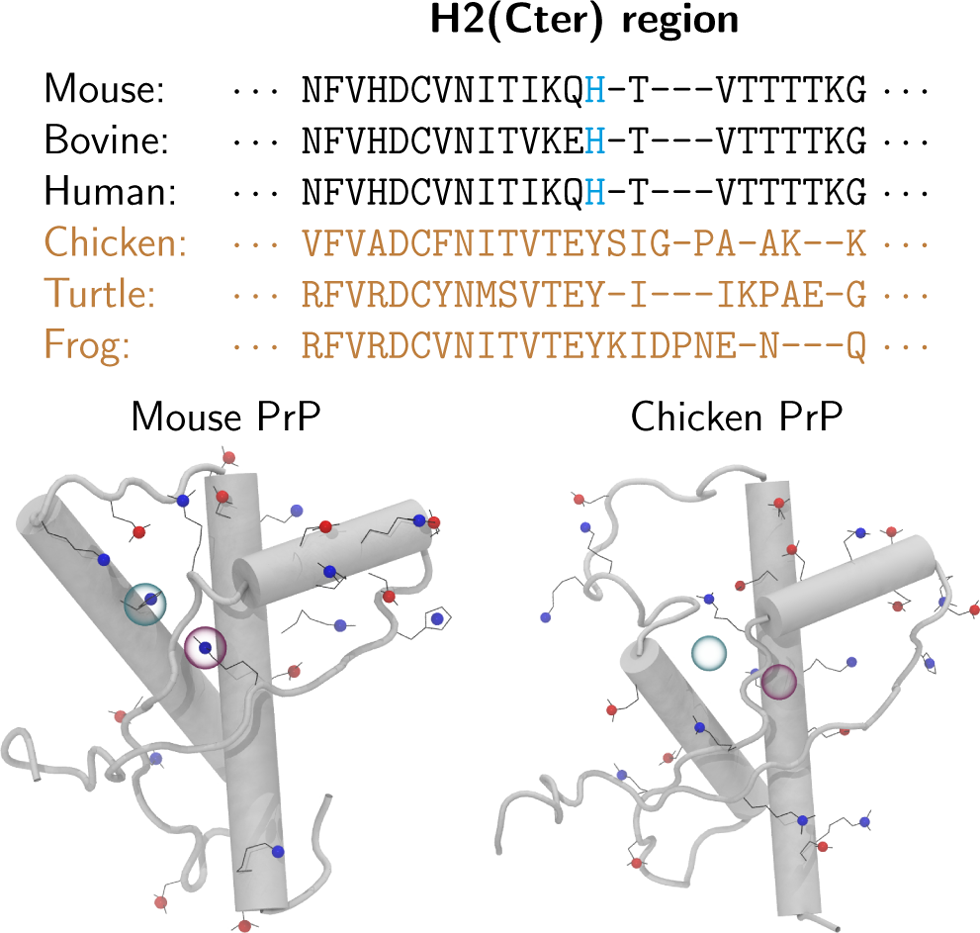

Supplement: Figure S4 — Mammalian VS non-mammalian species. The upper panel of the figure shows the sequence alignment in the H2(Cter) region. H187 is represented in cyan in the sequence and is conserved throughout all mammalian PrP. The lower panel represents the charged residues in examples of mammalian (mouse) and non-mammalian (chicken) PrP. The geometric center of positively and negatively charged groups are represented by blue and red opaque spheres, respectively. The two transparent spheres in cyan and magenta correspond to the position of and in mPrP, respectively. Sequence and structural alignments were done with the MultiSeq plugin [63] implemented in VMD [57]. (TIF) [file pcbi.1003057.s008.tif]

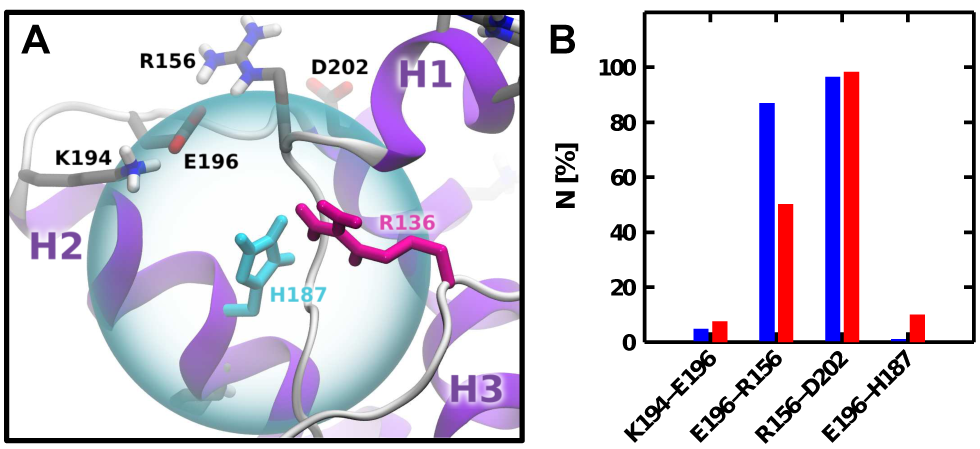

Supplement: Figure S5 — Charged residues around . (A) Relative positioning of the residues. All charged groups around fall approximately within the same range of distance, which is represented by a transparent sphere of 8 Å diameter centered on the geometric center of . (B) Population of salt bridges in our simulations with a neutral (blue) or protonated (red) H187. (TIF) [file pcbi.1003057.s009.tif]

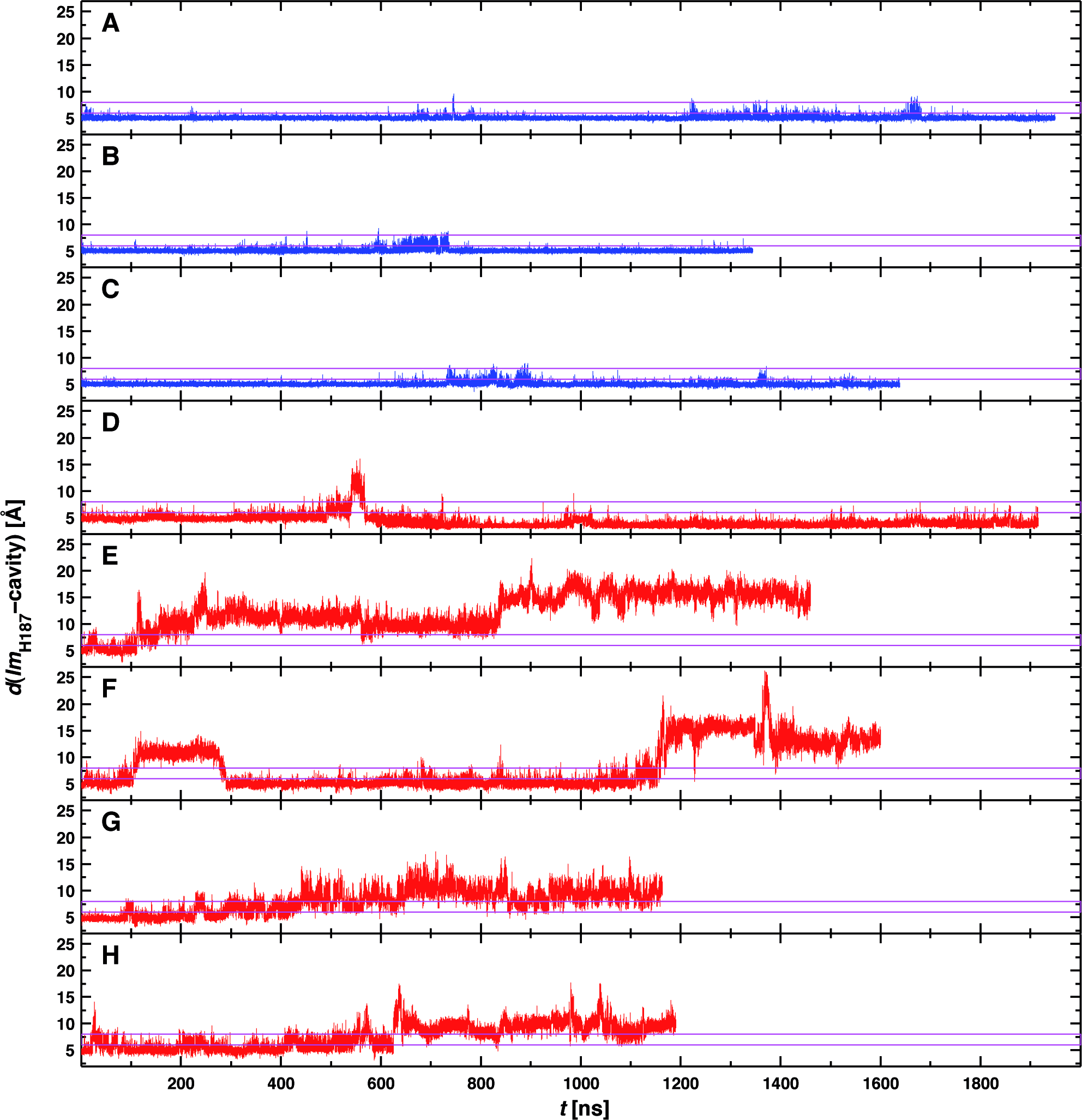

Supplement: Figure S6 — Distance of (or ) from its cavity as a function of time. (A–C) Three individual simulations in which H187 is neutral. (D–H) Five individual simulations in which H187 is protonated. The distance is defined as in Fig. 3-C. The magenta box represents the cutoffs used in Fig. 3-C to define , , and states. (TIF) [file pcbi.1003057.s010.tif]

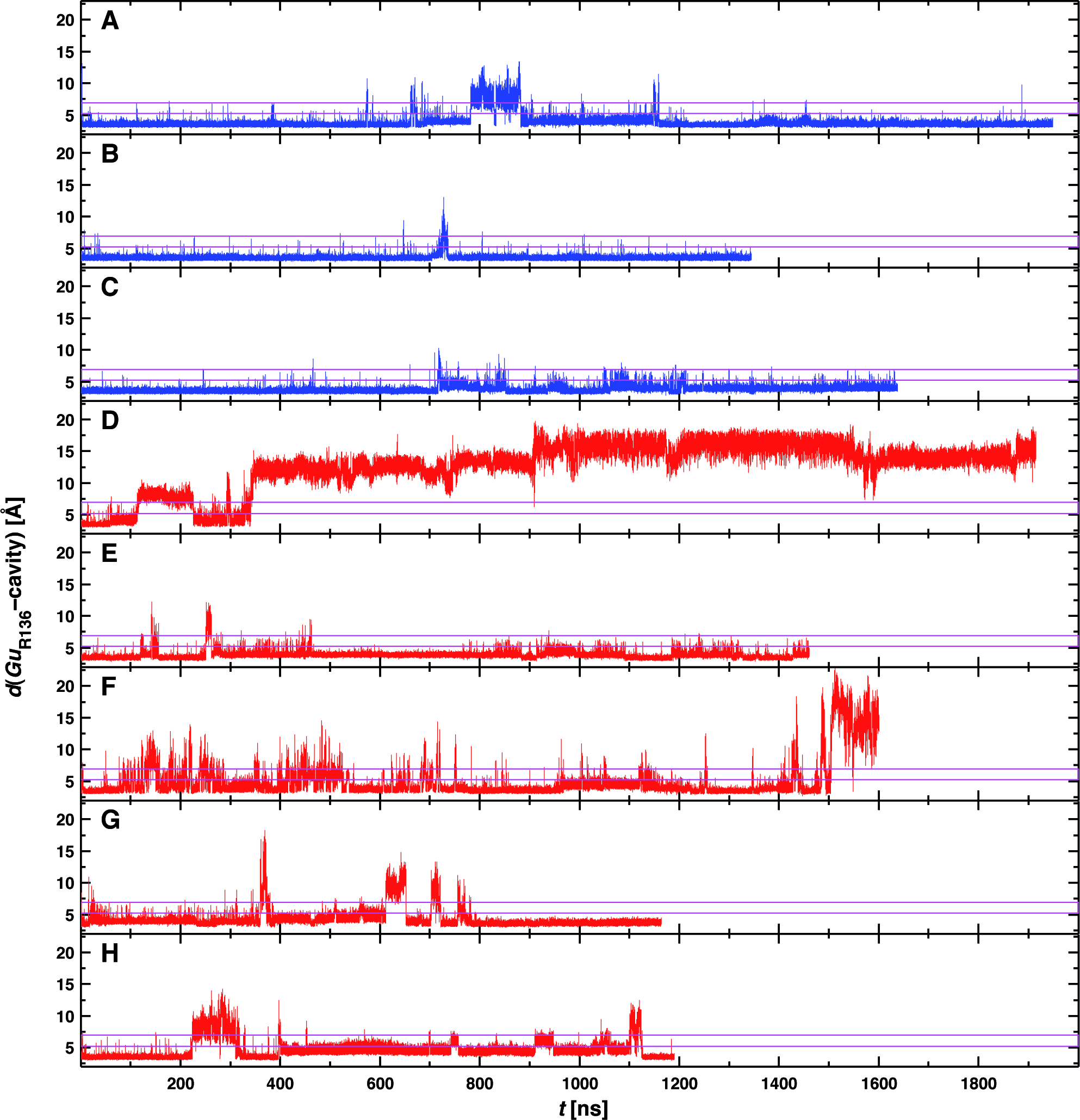

Supplement: Figure S7 — Distance of from its cavity as a function of time. (A–C) Three individual simulations in which H187 is neutral. (D–H) Five individual simulations in which H187 is protonated. The distance is defined as in Fig. 3-C. The magenta box represents the cutoffs used in Fig. 3-C to define , , and states. (TIF) [file pcbi.1003057.s011.tif]

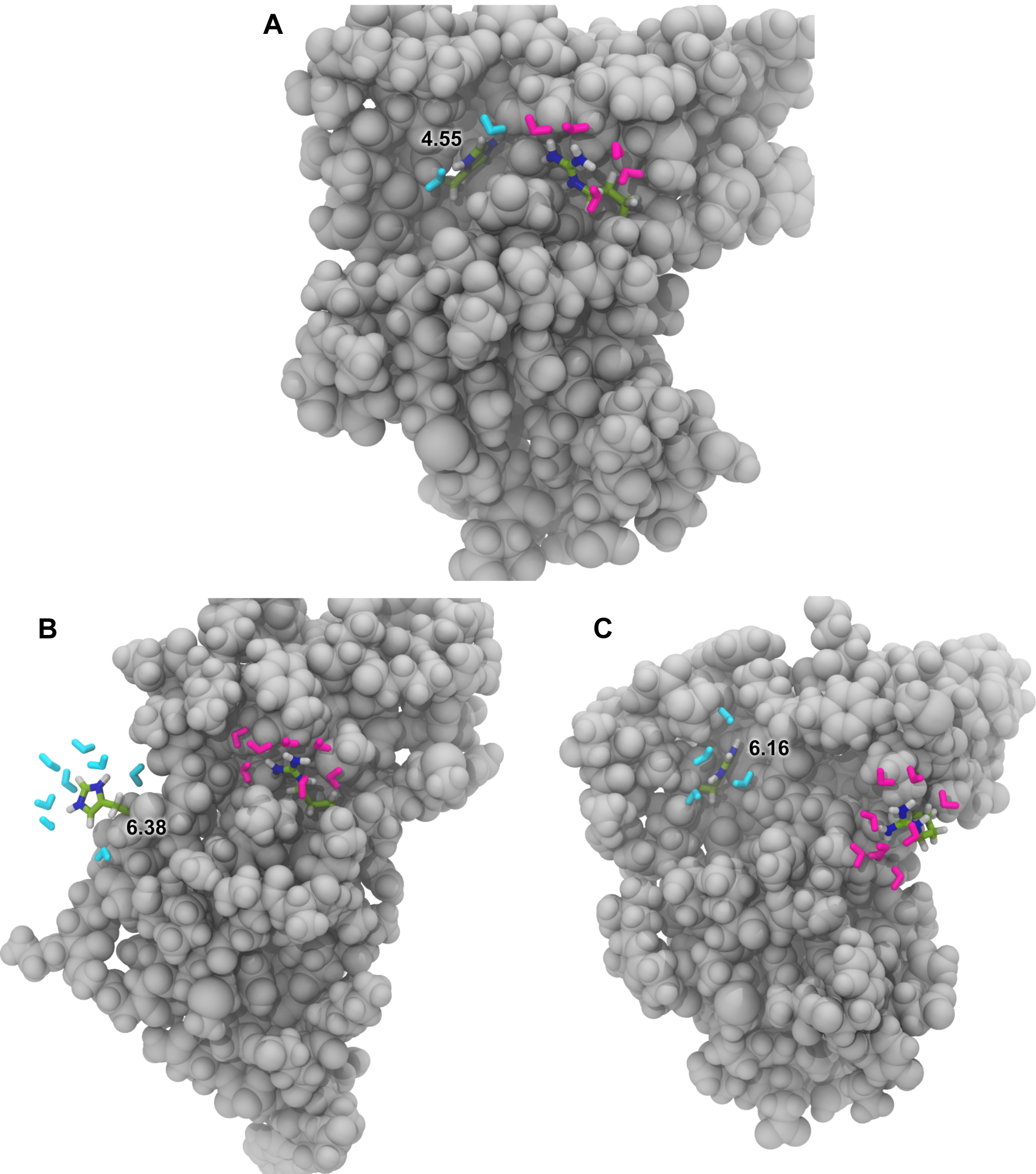

Supplement: Figure S8 — of H187 as a function of the relative positioning of and . Representative snapshots of (A) a state (equilibrated structure before or moves out of its cavity), (B) a state, and (C) a state. Water molecules that are within 3 Å of or are represented in cyan and magenta, respectively. The number close to H187 in each panel indicates the of this residue, as estimated by PROPKA [35] from the corresponding structure. The calculations were performed using the PDB2PQR software [64], [65]. We also provide the PROPKA output files corresponding to panels (A), (B) and (C) in Dataset S1, S2 and S3, respectively. (TIF) [file pcbi.1003057.s012.tif]
